# A qualitative study of the unique challenges faced by female police officers in Sweden

**DOI:** 10.1186/s12889-025-23839-1

**Published:** 2025-08-01

**Authors:** Frida Degerstedt, Sahibe Akpinar, Karin Fältman, Elin Granholm Valmari

**Affiliations:** 1 Community Medicine and Rehabilitation, Umeå University, Umeå, Sweden; 2 Vocational Rehabilitation for Employment, Swedish Public Employment, Linköping, Sweden; 3 Department of Rehabilitation Medicine, Skåne University Hospital, Lund/Höör, Malmö, Sweden

**Keywords:** Frontline services, Gender, Norms, Shift work, Work-life balance, Male-dominated profession

## Abstract

**Background:**

Policing has traditionally been viewed as a masculine domain, which may influence how the job is perceived. While female police officers currently constitute an increasing part of the workforce, policing remains a male-dominated field globally, and gender-related challenges persist. The current study explores how female police officers in Sweden navigate the gendered norms and physical challenges they face.

**Methods:**

Semi-structured and cognitive interviews were conducted with 11 female Swedish police officers. Reflexive thematic analyses, as described by Braun and Clarke, were employed to analyse the data. The methods and results were reported in accordance with qualitative reporting standards.

**Results:**

The findings are presented through one overarching theme: ‘Challenges with policing in a man’s world: Gendered expectations and physical realities’, describing how female officers in Sweden navigate physical and psychological demands in a profession designed for male bodies, finding creative solutions while facing ill-fitting equipment, physical strain, and challenges related to pregnancy and recovery. They also balance their roles as women, romantic partners, and mothers while managing the unique demands of policing and shift work. The results are separated into the following themes: ‘Fitting the female body and mind into a traditionally male profession’ and ‘The dual identity: A police officer and a woman’.

**Conclusion:**

Swedish female police officers navigate physical demands, gendered expectations, and work–life imbalance in a male-dominated profession. While resourceful in managing these challenges, the findings highlight the need for structural reforms. Even in gender-progressive contexts, women continue to face double burdens and unequal expectations. Hence, the findings serve as applicable both nationally and internationally, and promoting equity and inclusion is crucial for the well-being and sustainability of female officers.

## Background

Policing has traditionally been viewed as a masculine domain, which may influence how the job is perceived and managed by female and male police officers, respectively [1]. Although women now make up a growing share of the workforce, policing remains a male-dominated field where gender-related barriers persist [2]. A specific police culture reinforces these gender-related barriers, including a focus on emphasising virility, toughness, and masculinity [3], where the female body is suggested to have distinct disadvantages, just because it is classified as female and where the police culture tends to view men as having superior ways of using their weight and displaying endurance [4]. Furthermore, e.g., family life, intra-gender relationships, promotion process, and other physical limitations have been reported as challenges for female police officers [5].

In Sweden, women comprise 47% of the police force’s employees, including both police officers and staff working within the Swedish Police Authority. Among these, 34% are sworn police officers [6]. The number of female police officers in Sweden is higher than in many other countries [7]. However, despite this and the gender-equal opportunities and evolving police culture within the Nordic countries, the masculine police culture persists [8, 9]. For instance, within the Norwegian Police Authority, female police officers are discouraged from seeking special operations training due to the perceived cowboy masculinity and mythical information provided, despite them succeeding similarly to men when they have the courage to apply [10].

The police culture has been identified as particularly harmful to female police officers [11], who face specific challenges within this culture [12, 13].

Other challenges within the police force may include bullying and sexual harassment [14, 15] or being a token within the police force dedicated to domestic violence and body searching female suspects [4]. Female police officers have also been found to have fewer opportunities to advance to higher ranks [16] and inadequate mentoring [17]. Hence, Rabe-Hemp [18] reported that female officers performed gender while at work, i.e., the concept introduced by West and Zimmerman [19], where viewing gender not as something inherent to individuals, but rather as something that we perform in social interactions. For instance, by actively resisting as well as adopting stereotypical norms of femininity and policing, thereby broadening their work opportunities while reinforcing their traditional conception of gender differences [18].

Working as a police officer in frontline services requires navigating demanding contexts that can impact work-life balance and overall health [20]. Conflicts between work and personal life have been found to influence experiences of stress and burnout among police officers in general [20], and particularly among women [21]. A study by American researchers highlights the specific challenges faced by female police officers related to parenthood [22]. One contributing factor is that frontline police officers typically work rotating shifts, covering all hours of the day and night [23]. Shift work is associated with low job control and limited influence over work schedules [24–26]. It also negatively affects quality of life and leads to work spillover, making it difficult for officers to maintain boundaries between their professional and personal lives [27, 28]. Beyond work-life conflicts, shift work has broader health implications [29]. It has been linked to increased risks of mental health issues, sleep disorders, cardiovascular disease, and gastrointestinal problems [24, 30]. Night shifts, in particular, disrupt natural circadian rhythms, which can lead to fatigue during night shifts and disrupted daytime sleep [27]. According to a meta-analysis, approximately 50% of police officers experience poor sleep [31]. Thus, rotating shifts, particularly night shifts, can negatively impact the balance between work and family life, intensifying conflicts between professional responsibilities and personal commitments [32]. Additionally, balancing personal life with ongoing work obligations has been found to limit spare-time activities for police officers [33], leading to conflicts in work-life balance and, ultimately, increased stress [34].

Female police officers are particularly vulnerable to work-related stressors [35], which are further shaped by gender norms, such as household responsibilities. These norms may not only shape perceptions of a typical male profession but may also influence expectations around caregiving responsibilities and the ability to balance demanding work with personal life [36]. Norms are central to understanding structural inequalities [36], including those within the policing sector and the police culture. The term ‘norm’ may be defined in various ways, but in this study, it is defined as the collective definitions of socially approved conduct, rules, or ideals. Norms shape social structures by defining acceptable behaviour and guiding individual actions within groups. They are dynamic and negotiable, and individuals may hold multiple, sometimes contradictory, positions based on their various affiliations [37]. Gender norms play a vital role in these negotiation processes, as they influence power dynamics between men and women, where women are regarded as doing more household work both within actual households and on the labour market [38].

Women’s health at work remains underexplored despite evidence that biological and chronic health factors influence employment outcomes [39]. Within policing, a previous systematic review also identified gender as one of the most consistent risk factors for poor mental health [40]. In Sweden, gender-equal opportunities at work are a national policy goal (Ministry of Employment, 2016), and formal support structures are more accessible to female than male police officers. Nevertheless, female officers report inadequate workplace support [41]. Thus, female police officers still seem to face gendered challenges, despite Sweden’s nationwide gender equality policies and a strong emphasis on gender-equal working life. As well as being more represented than in many other countries. Hence, this study aims to explore how female police officers in Sweden navigate the gendered norms and physical challenges they face.

## Methods

### Study design

The study employs an experiential qualitative framework to gain insight into the participants’ perspectives. It is an explorative design, where our fundamental approach is based on interpretivism, which involves gathering knowledge from participants’ personal experiences and interpreting the underlying meaning of their subjective realities. We then connect these experiences to gender theories and theories of human behaviour, which have also influenced our understanding of the data. Hence, reflexive thematic analyses, as described by Braun and Clarke [42], were employed throughout data collection until analysing and writing the manuscript [43]. The current study employed semi-structured and cognitive interviews to gain an understanding of the experiences of female police officers.

The methods and results were described in detail according to a qualitative checklist. We used the Qualitative Design Reporting Standards (JARS-Qual) [44] to ensure the transferability of our findings. The JARS-Qual was used for data collection and dissemination of the results. The Consolidated Criteria for Reporting Qualitative Research (COREQ) [45] was also attached as part of the journal’s submission process. Ethical approval for this study was obtained from the Swedish Ethical Review Authority (Reg. No. 2022-03690-02), which follows the Helsinki Declaration [46]. For instance, the research project was explained to the participants, information was provided both orally and in writing, and written consent was acquired via an ‘approval to participate’ form.

### Participants

The inclusion criteria were police officers in frontline services who identified as female. In total, the data includes 11 women, with ages ranging from 28 to 54 years. They worked in different police regions across Sweden, in both urban and rural areas. The participants had between three and thirteen years of experience working at the Swedish Police Authority. Most were in their late 20 s or early 30s. Seven participants had one or more children living with them at home, while one participant had grown-up children. Eight participants were cohabiting with their romantic partners, while the rest were single or not cohabiting with their partners. The participants mainly worked within frontline services or were temporarily working elsewhere within the Swedish Police Authority. Some participants also had leadership experience within the Swedish Police Authority.

### Data collection

The data are collected at two different time points, as part of two separate data collections within a larger research project exploring the life balance of Swedish police officers. The data were first collected between February 2021 and August 2021 with semi-structured interviews, and then between January 2023 and May 2023 with cognitive interviews.

The same sampling strategies were used for both data collections. First, posters, social media, and personal contacts within the Swedish Police Authority were utilised to disseminate information about the study, which employed convenience sampling. Police officers were asked to spread the study information, even if they were not eligible to participate themselves. Then, snowball sampling was used, followed by purposive sampling, to achieve participant variation and diversity. Six women were interviewed via semi-structured interviews, and five women were interviewed via cognitive interviews. After the last five interviews were added to the dataset, information power [42, 47] was considered to have been reached due to the richness of the dataset, and the author group decided not to include any further participants in the dataset.

The last author created the interview guide for the semi-structured interviews. It was organised into topic areas reflecting aspects of a person’s lifestyle, health-related questions regarding physical and mental health, and how life, both at work and in private life, is experienced and balanced. Braun and Clarke’s method for allowing theories to influence the interview guide [42] was utilised. Hence, theories from occupational science were used to create the semi-structured interview guide [48–50]. See Table 1 for a short description.

For the cognitive interviews, questions were based on the same topics as the semi-structured interviews. However, they were phrased as survey questions, and participants were asked to rate the questions, followed by verbal explanations for their ratings as they reflected on examples from their everyday life.

**Table 1:** Interview topic guide

Topic guide The semi-structured interview guide covered the following topic areas in the different lives of female police officers, adding follow-up questions as needed depending on the answers and individual characteristics of the specific person being interviewed: • Life roles and activities as experiences both at work and in private life, how these are balanced, and how they are influenced by the social and physical environment at home and at work. • Values, meaning, engagement, and competences both at work and in private life. • Health-related questions regarding physical, mental, and social health.	

All interviews were conducted in person or via visual feed, such as using Zoom or FaceTime, depending on the participants’ preferences, and lasted on average between 1 and 2 h. The last author conducted and audio-taped the interviews. Professional transcribers, the last author, and a medical secretary transcribed the interviews. For confidentiality, identifying details were omitted or altered.

#### Data analysis

A six-phase, reflexive thematic analysis, as described by Braun and Clarke [42], was employed. This includes back-and-forth movement between the steps and exploring the pattern of meaning across the dataset [42]. All authors participated in the analyses throughout all phases and have reviewed the transcripts multiple times. First, the second and third authors coded the semi-structured interviews. The first and last authors coded the cognitive interviews. The authors employed an inductive line-by-line coding process to analyse both the semantic and latent meanings in the data. Then, the data and codes were merged in a qualitative analysis software, MAXQDA [27], while creating clusters by the last and first authors. The second and third authors triangulated the entire dataset by reading the transcripts and identifying similarities, paradoxes, and controversies across the dataset. Clusters were then discussed among all authors several times, with the process involving repeated back-and-forth movement between transcripts, coding, and clusters. Once there were distinct contrasts between clusters, including a central organising concept, candidate themes were developed from the data.

Themes were developed based on the content of the data, where themes were generated from the data during our inductive process. They were refined by all authors, with the last author collecting quotes to illustrate the grounding of the data in the themes. All authors reviewed the final analysis, ensuring that quotations within a theme were consistent with the theme itself. Each author has contributed specific knowledge to the analysis. The first and third authors are registered physiotherapists, with the first author having a particular understanding of the intersection of gender and health. The second and last authors are registered occupational therapists, with the last author having particular knowledge of the health of police officers. As Braun and Clarke [42] emphasise the importance of theories during data collection and data analysis, it should be noted that gender theories [36], theories on human behaviour such as from occupational science [49, 50], as well as theories of biomedical health [51] have been used throughout the study.

## Results

The findings are presented through one overarching theme: *‘Challenges with policing in a man’s world: Gendered expectations and physical realities’*. This theme captures how female officers navigate the police culture, shaped by masculine norms, where not only the physical environment (e.g., uniforms, equipment, shift work) but also the cultural expectations of policing remain gendered. Participants described how their professional lives were interwoven with expectations of physical strength, emotional containment, and unlimited availability, expectations that align more closely with traditional masculine ideals than with the realities of many women’s lives, especially when caring for children.

**Fig. 1:**
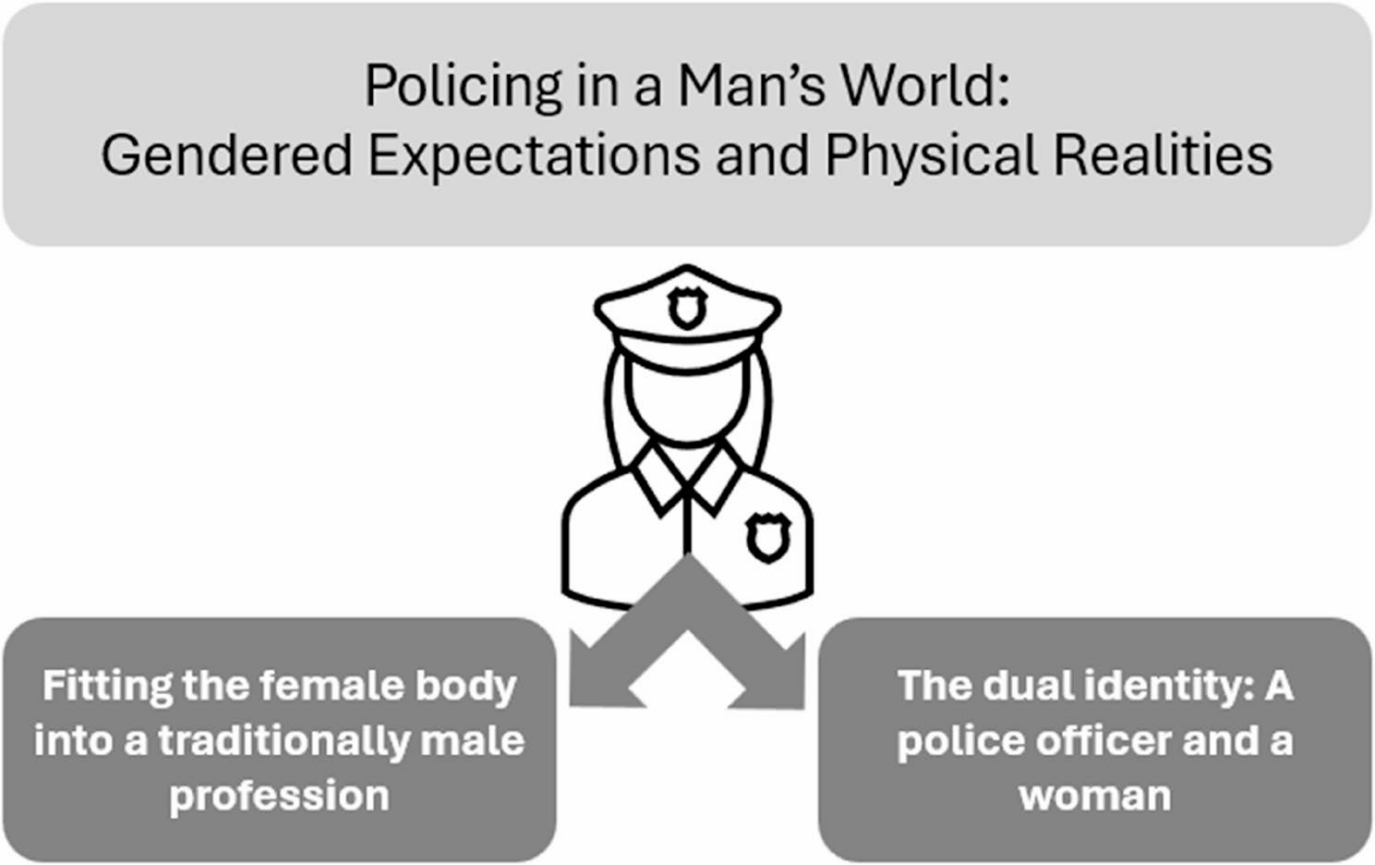
Description of the overarching theme and the two themes

The gendered expectations also extended beyond the workplace. Participants found themselves doing most of the unpaid domestic responsibilities, organising and coordinating family life in ways that reinforced traditional gender roles. Being a police officer and a woman often meant living a ‘double shift’, where juggling demanding, inflexible work with caregiving duties that remained socially expected of them as women. Female officers also described how motherhood clashed with professional norms, and how pregnancy could delay promotion, and parental obligations. Gendered expectations, thus, manifested both structurally and socially. Despite these challenges, the female police officers in this study also demonstrated creativity, carving out professional identities and coping strategies within, and in tension with, the system. These complex experiences are further examined in the following two themes: *1: ‘Fitting the female body and mind into a traditionally male profession’*, and *2: ‘The dual identity: A police officer and a woman’ (see* Fig. 1*).*

### Theme 1: fitting the female body and mind into a traditionally male profession

This theme examines how female police officers navigate the physical and psychological demands of a profession with masculine ideals designed for male bodies, including ill-fitting equipment, chronic physical strain, and challenges related to pregnancy and recovery.

Participants described how gendered expectations often aggravated their struggle to balance professional responsibilities with personal roles, resulting in guilt and stress. Despite these challenges, the participants showed resourcefulness by utilising strategies such as communication, adaptability, and prioritisation of recovery to manage their roles while emphasizing the need for systemic changes to better support their unique experiences.

Due to the nature of their police job descriptions, the participants faced various demanding issues arising from the physical requirements and stressful environments of their roles. Participants who were physically smaller or perceived themselves as insufficiently strong described how they utilized communication to navigate threatening situations differently than many of their male colleagues when interacting with criminals and potentially violent individuals. This strategy helped them avoid being perceived as threatening, which was identified as an advantage over more confrontative methods used to a higher degree by their male counterparts. The participants emphasised how a respectful approach often elicited respect in return, even during encounters with individuals who had committed crimes. One participant explains it like this:

*“I’m not two meters tall and weigh 100 kilos. I mean…I use my mouth*,* and many criminals…most of them are men*,* and they don’t get as provoked by me. They don’t need to measure up…if they were to pick a fight with me… it wouldn’t show strength because I’m like half their size…"*.

*(Police officer 6)*.

Another challenge raised was the experience of physical discomfort due to their work equipment. Despite adjustments made to accommodate women, the equipment was not fully designed to fit the female body and its specific needs, leading to discomfort, pain, and physical strain. The heavy equipment can cause long-term physical issues, which are exacerbated by poor posture and prolonged sitting in patrol cars. The design of the car seats and the weight of the equipment pressing against the body further worsened the situation. Participants noted that protective vests and trousers were poorly fitted for women, with the vests pressing against their chests. One participant even removed a chest plate to accommodate her breasts, compromising her safety. Health issues reported by the participants included lower back and leg pain, as well as stomach pain and bloating, which some participants attributed to the overall workload and shift work. The weight distribution of equipment belts was uneven due to women’s generally smaller waists and wider hips. This puts the body under irregular strain, causing pain in the hips, neck, and shoulders. Some participants had opted to wear a specialised utility vest instead of a belt to reduce strain. However, at times, this was more of a compromise than a relief, as it was reported to strain other parts of the body, as explained by a participant returning to work after pregnancy.

*“I mean*,* the equipment is so heavy. And especially when you come back after a pregnancy*,* your body is so fragile. And I wasn’t in the same physical shape at all… After every shift*,* my sciatic nerve got pinched. And then I had to…wear a vest instead. But then all the weight ended up on my chest and stomach*,* so I got exhausted in my shoulders instead…"*.

*(Police officer 1)*.

Therefore, policing posed unique challenges both during and after pregnancy. For instance, their roles and physical capabilities changed significantly, and the lack of adjustable uniform sizes restricted both mobility and comfort during shifts. Furthermore, beyond the physical aspects, there was also a psychological dimension to consider regarding being female, especially in terms of pregnancy. Some participants felt pressured to compromise their safety and well-being to avoid being reassigned to desk duties when they became pregnant, as they perceived it to be boring. Although some attempted to remain in frontline services as long as possible, concerns were also raised about the risks of injury from violent encounters, which led to stress and, at times, feelings of guilt regarding their unborn child, as illustrated below:

*“… with my first pregnancy*,* I had someone lunge at me with a knife*,* and it was only afterwards that I reflected…shit*,* what if it had hit my stomach.”*

*(Police officer 6)*.

There was also an increased awareness of infection risks during pregnancy, which became particularly evident during the coronavirus pandemic. This heightened awareness of risks also influenced their working style, contributing to increased psychological strain. For instance, one participant described how indirect threats from individuals who had been apprehended led to her decision not to live near work, in order to limit the risk of encountering these individuals in her personal life. As a woman, she felt vulnerable in her private life because she did not have her uniform on, especially since she was aware of the risk of violent encounters if she was recognised.

Physical ailments influenced their work and their ability to engage in leisure activities and social life. This was particularly true for those suffering from chronic conditions such as pain and inflammation in their knees and backs, which hindered their ability to stay active or fully participate in family life. Some participants reported hesitancy to admit to having health issues; instead, they endured pain and discomfort. Regular health check-ups were also encouraged. Nevertheless, the responsibility for preventing or addressing work-related health problems often falls on the individual. Many participants noted that discipline, job demands, and time were critical factors affecting their ability to maintain a regular exercise routine.

*”…sometimes it’s midnight because I’ve put the kids to bed and done the dishes and yeah*,* you know… And then it just has to be done then (the exercise).”*

*(Police officer 2)*.

Hence, despite most workplaces offering opportunities for exercise during working hours, this was not perceived as sufficient and finding time to exercise became even harder when raising children.

### Theme 2: the dual identity: a police officer and a woman

This theme highlights how female police officers navigate the intersection of their roles as women, romantic partners, and mothers with the unique demands of policing.

Participants without children reported fewer challenges in managing work-life balance, as they could prioritise their own needs, such as rest and recovery after night shifts. For those with children, family life took precedence, often requiring sacrifices in personal time, finances, and career progression. One participant explained the differences between her and her colleagues who have children:

*I’m a bit spoiled in that way, since I don’t have kids myself or anyone else I have to adjust to…I can sleep as long as I want. I can allow myself that, and so I do*.

*(Police officer 5)*.

Parenting as a police officer was particularly demanding due to the challenges of shift work, overtime, and job unpredictability. Participants with young children described how managing parenting responsibilities, such as preschool routines and social activities, alongside irregular schedules often led to feelings of guilt about not having enough time or energy to meet their own or their children’s needs. Maintaining stable routines proved difficult, frequently requiring last-minute childcare arrangements.

There was a consistent belief that physical activity is a crucial part of life, essential for handling both work-related and personal challenges. For example, exercise can help prevent injuries, enhance overall well-being, and promote mental and physical relaxation. However, several participants encountered various obstacles in establishing a regular exercise routine, including a lack of time and energy. Some participants tried to integrate physical activity into their daily routines, such as walking their dogs, exercising with their children, or cycling to work. While some were able to maintain a workout routine, common sentiments expressed in the findings included a desire to exercise more frequently or at a higher intensity, along with a feeling that exercise was often deprioritised, especially after having children.

Being the primary caregiver or navigating shared custody arrangements added another layer of complexity, making flexibility in scheduling essential. While some participants sought more predictable daytime roles to better manage their family responsibilities, others highlighted the benefits of shift work, such as spending time with their children during the day. However, these benefits were also described as limiting sleep time and recovery after night shifts. There were also voices highlighting how women tend to remain in investigative roles after being transferred from frontline services during pregnancy. One participant explained how traditional family responsibilities were perceived to be at the disadvantage of the mother in heterosexual relationships, particularly if both work as police officers:

*“… often*,* there are quite a few police couples*,* and when a baby comes along*,* it’s usually the woman who moves on to investigations*,* and the man stays in frontline duty*,* including shift work…”*.

*(Police officer 4)*.

Participants assigned to desk duties during pregnancy also faced a decrease in income due to the loss of unsociable hours allowances and delays in career advancement. One officer further explains the differences she perceived between men and women at work, detailing why men tend to advance more quickly in their careers:

*“…we women are often a bit more self-critical and don’t quite believe in ourselves as much…men often take on responsibility earlier… But women generally need a bit more support in that… So*,* I would say it does have an impact*,* even if people try to say that it shouldn’t.”*

*(Police officer 9)*.

However, these sacrifices for career progression provided a better work-life balance, which was described as crucial for maintaining long-term careers. Access to flexible scheduling was essential for managing family and work life, with participants noting that supportive supervisors and individualised solutions offered relief. Nevertheless, not all participants felt they received sufficient organisational support. Some emphasised that systemic improvements, such as reducing consecutive night shifts, could alleviate stress related to managing work-life balance. Moreover, implementing nighttime care for children, as one participant suggests:

*”I still think that if the possibility existed*,* maybe more people could stay in frontline services. It (the awareness) just needs to be raised somehow—that the employer…values frontline services.”*

*(Police officer 3)*.

Still, the perceived stress at work from repeatedly adjusting their hours, such as picking up sick children from daycare, appeared to be internalised, as colleagues were described as supportive and understanding. The strain of balancing professional and personal responsibilities also appeared to have a negative impact on participants’ health and well-being. Many described feeling fatigued and exhausted, struggling to recover after shifts, and lacking the energy for social or leisure activities. Additionally, when asked if feeling stressed about not knowing when to get off shifts, a police officer working three shift rotations answered:

*”Only if I’m*,* like*,* driving the kids to figure skating or choir practice. Then it can be like… hey*,* now I have to take a break. I have to sort things out. I have to arrange with some neighbours…They (colleagues) do get annoyed*,* but most are still understanding…it’s stressful. It is. Especially when it’s that close in time.”*

*(Police officer 11)*.

This often led to irritability and emotional strain for the police officers. To cope, participants relied on support from family or partners, developed specific routines for sleep and recovery, or sought solutions such as adjusting their shifts to minimise disruption to their private lives. However, the cumulative effects of unpredictability and shift work challenges sometimes raised questions about whether policing was a sustainable long-term career. Some described making sacrifices in both their careers and personal time for the sake of their families. For separated parents, the demands of shift work and custody arrangements required careful planning to ensure time with their children, often at the expense of personal needs or hobbies. Despite these challenges, participants expressed a strong sense of fulfilment in their dual roles. The sacrifices were described as worthwhile, framing participants’ lives as meaningful and rewarding despite the trade-offs.

The division of household responsibilities added another layer of complexity, with participants often acting as the ‘project leader’ at home, managing both visible and invisible tasks. This imbalance occasionally led to conflicts, and some participants noted that relationship breakdowns were partly attributed to the stress of unevenly distributed responsibilities.

*“…I mean*,* we have it more equal than many others. But I wouldn’t say it’s completely equal…For example*,* with the kids*,* I handle everything. But it’s also a bit about how I am as a person*,* you know? I’m not sure; I feel very strongly about my kids*,* just like everyone else does.*

*(Police officer 6)*.

Therefore, this participant took on more responsibility for the home and children than her partner and accepted this unequal division. Such inequities contributed to participants’ exhaustion, and in terms of their careers, benefit the partner who assumes less responsibility for family life, allowing them more time and energy for work and self-care. Gendered inequalities were also noted during work hours, where men were viewed as supporting each other in ways that left female officers more vulnerable due to a lack of collegial and professional support.

*“There is… I usually call it ‘the boys’ mutual admiration club’*,* like they pat each other on the back… If you bring in a woman who disagrees*,* especially if you’re also significantly older and more experienced. Then it gets tough.”*

*(Police officer 10)*.

Traditional gender roles appeared to influence the work-life balance of female police officers. Nonetheless, the participants primarily emphasised the significance of workplace flexibility, fair policies, and supportive colleagues in aiding them to navigate the dual demands of their professional and personal lives.

## Discussion

This study examines how Swedish female police officers navigate gendered norms and physical challenges in their profession. Officers faced significant discomfort due to equipment and uniforms designed for male bodies, with pregnancy further exacerbating these issues, creating safety risks and financial setbacks. Beyond work, traditional gendered household responsibilities, combined with demanding schedules, intensified emotional and logistical burdens. Despite these obstacles, female officers demonstrated resilience, utilising effective communication and emotional intelligence to navigate challenges. While these skills were considered more effective than physical force, they were still shaped by the profession’s masculine norms.

### Physical demands in male-dominated professions

The physical strain caused by poorly fitted equipment and uniforms designed for male physiques is compelling, considering the increasing percentage of women in the Swedish police force [6]. According to Malbon et al. [52], the primary areas where body armour either rubs or causes discomfort for female officers are the left and right anterior mammary regions and the posterior lateral sacral region. By understanding bra size distribution, the type of bra worn, and areas of discomfort, improvements in body armour design could and should be developed [52]. However, our study indicated that the burden of adapting to the structural inadequacies of uniforms and equipment seemed to fall on the participants themselves, who had to seek individual solutions, such as modifying their equipment. Orr et al. [53] reported that upper-body and trunk endurance, lower-body power, and aerobic fitness are essential for preparing female police officers to perform their occupational duties [53]. Ideally, this should be facilitated during work hours. Nevertheless, our results reveal that female officers are reluctant to disclose experienced pain and instead endure it. The internalisation of the cause of pain, such as believing they have not exercised sufficiently to cope with strain, could contribute to this hesitancy, primarily if the norm of strength and endurance is based on male standards, thereby pressuring more females to live up to it. Furthermore, a previous review of police officers has highlighted that musculoskeletal pain is associated with psychosocial stress, emphasising the importance of addressing psychosocial risk factors to enhance the overall quality of life for police officers [54]. Female police officers have been reported to experience more stress compared to their male counterparts [14, 40]. Given the additional gendered challenges faced by the participants in our study, leadership and social support are vital factors to consider when addressing police officers’ musculoskeletal pain, as previously discussed by Granholm Valmari [55].

Our findings highlight the gendered inequities within police organisations, where structural systems have not evolved to support female officers’ physical and professional needs fully. This can also be observed in other Nordic countries, such as Iceland [13] and Norway [8, 10], where gender equality is high on a national level [56]. Furthermore, Treece [57] found that gender inequality was one reason for job dissatisfaction among female officers. According to Murray [58], female officers describe experiences of workplace sexism and deploy adaptive strategies daily in their workplaces to resist gender inequality. In the same study, both men and women described the police profession as masculine, with men being the ideal police officers. The existing “old police culture” and “old boys’ club” were criticised by Murray [58], and this phenomenon was also observed in our results, where men were perceived to support one another, leaving female police officers out. Moreover, our study found examples of how pregnancy introduced unique challenges, as female officers faced not only physical discomfort but also career-related consequences. The transition to desk duties during pregnancy may be perceived as a privilege and adaptation to an increased vulnerability, as there are indications by previous research on police officers that shift work increases the risk of pregnancy loss [59]. Nevertheless, these adaptations could also lead to undesired financial losses and delayed career progression.

The participants in our study also demonstrated resourcefulness in navigating the presumed challenges of being a female officer, for instance, by utilising effective communication and emotional intelligence in high-pressure situations. This aligns with Rabe-Hemp [18], who found that female police officers differentiate themselves from their male counterparts by both performing gendered roles and engaging in police work. The creativity of female police officers in adapting to the police profession, as highlighted in this study, also underscores the importance of non-physical skills in the policing profession. It emphasises the distinctive contributions that female officers make to the field of policing. Shoub et al. [60] exemplify this by revealing that female police officers have lower vehicle search rates but confiscate the same net amount of contraband as their male counterparts. Thus, it minimises the number of negative interactions with citizens without compromising effectiveness [60].

### Work-life balance and gendered expectations

While work-life balance issues are common to both men and women, they are also present within a policing context [33, 61]. Previous research on police officers has found indications that female officers experience unique challenges within the police organisation [5]. Examples include experiencing burnout due to handling violent arrests or the potential use of force. This might indicate a double burden, as well as a triple burden issue for female officers belonging to cultural minorities, where predominantly minority female officers have been found to have an even higher risk for burnout [62]. Our study highlights a double burden faced by female police officers, balancing traditionally gendered household responsibilities alongside the physical and mental demands of their profession. Participants described themselves as their family’s ‘project leader,’ managing most logistical and caregiving tasks. This greater overall responsibility, compared to their male counterparts, often led to emotional strain, feelings of guilt toward colleagues, and logistical challenges in their private lives, especially when compounded by unpredictable shift work and overtime. Rocha et al. [34] found that having young children, older adults, or other dependents at home, combined with shift work, negatively impacts the work-life balance perceptions of female police officers [34].

The competing demands of work and home life also left some of the female police officers in our study feeling exhausted, with limited time or energy for recovery or leisure activities. While feeling exhausted and having a lack of time is not gender specific, earlier research comparing women and men indicates that women experience a double burden, causing more life imbalances compared to men [63]. Hence, gender-specific health interventions might be needed to mitigate the risks of ill health for female police officers. According to Chitra and Karunanidhi [64], resilience training for female officers is effective in enhancing job satisfaction, reducing work stress, and improving mental health among female police officers [64]. Moreover, the importance of organisational support was reported by Illias, Demou, et al. [65], particularly in promoting mental health and facilitating a return to work after parental leave, as these constituted particular risks for female officers who are also mothers [65]. Furthermore, team support and a favourable diversity climate are crucial in helping female officers navigate the potential conflict between their gender identity and their professional role within police organisations [66].

The impact of the dual burdens that female officers experience appears to extend beyond work-life balance, affecting the career trajectories of female police officers as well. A previous study found that organisational injustice influences the health and well-being of female officers, as well as their career progression and work-life balance [67]. Organisational injustice includes four modules: Procedural injustice referring to recruitment and retention of female officers, and bureaucratic and structural obstacles; relational injustice refers e.g. to leadership inequality, macho attitudes and internalised as well as externalised sexism; distributive injustice includes tokenism and deprivation of resources, and gendered injustice includes some of the relational issues such as normalisation of sexism, but also stigma towards reproductive events and mental illness [67]. In our study, pregnancy and caregiving responsibilities after birth were cited as reasons why female officers transitioned out of frontline services or sought fewer demanding roles within the police organisation, thus not only prioritising family but also prioritising their male partners’ careers before their own. The findings reflect broader gendered inequalities within the workforce, even in Sweden, where the percentage of female police officers is considerably higher than in many other countries, and the number of female officers continues to increase [7]. Hence, the present police culture of masculinity still dominates, and women disproportionately bear the cost of balancing professional and personal responsibilities. Results are in line with previous research [68], which found that female police officers who achieve seniority at work often share similar career experiences as male officers, especially at higher ranks. These experiences included long hours and prioritising work over family. Hence, a career generally comes at the cost of deprioritising other parts of life. However, the social costs tend to be higher for female officers as women are traditionally socially rewarded for caring family practices. In contrast, family sacrifices entail more severe social and professional consequences for women than for men. Thus, women’s greater costs to advancement compared to men affect their progression in leadership [68]. In conclusion, career sacrifices may be perceived as worthwhile when prioritising relations with family and children and one’s health, but they also entail frustration. Zikic [69] agrees that career sacrifices may entail satisfaction when perceived as being for the greater good, but also acknowledges regrets. Our results reveal how gender appears to influence career sacrifices and the extent to which they are accepted or expected.

## Strengths and limitations

A few limitations should be mentioned. First, the study should acknowledge the small sample size, which was due to the difficulties in recruiting participants. Thus, as indicated by previous research on the complex challenges faced by women in the police force due to the masculine culture of policing, some prior challenges were not found in this study [5]. To mitigate limitations in terms of credibility and transferability due to the small sample size, two separate data collections were conducted, employing diverse methods for data collection. This may have implications for the study’s transferability. Although limited to a small sample size, the participants’ diversity in terms of age, professional roles, family situations, and geographic locations across Sweden, including both urban and rural settings, enhances the transferability of the findings [45]. This enabled an in-depth understanding of a complex phenomenon, particularly in an under-researched population, such as female police officers. However, including intersectional challenges associated with, for example, ethnicity, sexuality or sexual identity [58] remains to be explored within a Swedish context and warrants further study.

Second, a strength of this study is its inductive analysis of both latent and semantic interpretations, which enables a nuanced exploration of the professional and personal experiences of female police officers. However, qualitative research introduces the potential for subjectivity. Hence, reflexivity becomes essential [42], and we should acknowledge our different theoretical underpinnings, which have been incorporated into the analysis, such as gender theories and theories of human behaviour and health. The interviews were conducted by the last author, an occupational therapist with extensive knowledge of police officers' health, which likely facilitated rapport and open discussions while reducing the need for participants to explain job-specific details. However, this familiarity may have introduced interviewer bias. The healthcare training of the research team likely also facilitated the analysis of the data with their holistic view of health and rehabilitation. However, due to our diverse experiences and theoretical backgrounds, we regard this as a strength of the study, especially during triangulation, where all authors collaboratively reviewed the coding process to ensure analytical consistency and enhance the study’s credibility, aligning with principles of methodological rigour [44]. Awareness of our reflexivity was integral to mitigating potential biases.

Lastly, combining the use of both semi-structured and cognitive interviews may provide both strengths and limitations to this study. However, the first data collection did not provide sufficient richness to the dataset. In contrast, our second data collection provided us with information power, in line with Braun and Clarke’s methodology for reflexive thematic analysis [42, 70]. While integrating data generation methods may be challenging for analysis, the datasets were analysed separately before integration. Furthermore, iterative data analysis, along with researcher triangulation, was employed to strengthen the credibility of the thematic development further, as emphasized by qualitative standards [44]. However, combining data also enriches the dataset by capturing both the depth of lived experiences and participants’ reflections on the interview process.

In summary, in line with the suggestions of Nowell et al. [71], to meet the criteria of trustworthiness for our study, we have triangulated all phases of our analysis, from phases 1 through 6, during our reflexive thematic analysis. For instance, we have kept reflexive thoughts from the data collection phases, as well as well-organised records, notes, transcripts, and reflexive journals. We have employed triangulation throughout all stages of the analysis and have also conducted regular debriefings between the authors, achieving consensus at every stage. We have also described our theoretical, methodological, and analytical choices throughout the different stages of the study.

## Conclusion

This study highlights the complex challenges that Swedish female police officers face, including physical demands, gendered expectations, and work-life balance in a traditionally male-dominated profession. Internationally, the findings contribute to the growing body of research on gendered experiences in policing, underlining that even in gender-progressive contexts, women in male-dominated professions continue to navigate double burdens and unequal expectations. Thus, the Swedish case serves as an illustrative example that formal equality does not automatically translate into lived equality in everyday professional life. Moreover, while the female police officers demonstrated resourcefulness in managing gender norms, the findings underscore the urgent need for structural reforms to support female officers. By addressing inequities and creating an inclusive environment, police organisations can enhance employee well-being, prevent ill health, and build a more sustainable police force. This includes, for instance, redesigning uniforms and equipment to accommodate different body types within the police force, promoting flexible solutions for parents, especially women, to avoid female officers being assigned only to desk duties after having children, and ensuring equitable promotion and career development opportunities following pregnancy.

## Data Availability

No datasets were generated or analysed during the current study.

## Abbreviations

COREQ: Consolidated criteria for reporting qualitative research
JARS: qual–Journal Article Reporting Standards for Qualitative Research
